# Integrated QTL mapping, gene expression and nucleotide variation analyses to investigate complex quantitative traits: a case study with the soybean–*Phytophthora sojae* interaction

**DOI:** 10.1111/pbi.13301

**Published:** 2019-12-06

**Authors:** Maxime de Ronne, Caroline Labbé, Amandine Lebreton, Humira Sonah, Rupesh Deshmukh, Martine Jean, François Belzile, Louise O’Donoughue, Richard Bélanger

**Affiliations:** ^1^ Département de phytologie Université Laval Québec QC Canada; ^2^ Institut de Biologie Intégrative et des Systèmes (IBIS) Université Laval Québec QC Canada; ^3^ CEROM Saint‐Mathieu‐de‐Beloeil QC Canada

**Keywords:** *Phytophthora sojae*, soyabean (*Glycine max* (L.) Merr.), QTLs, RNA‐seq, SNPs, horizontal resistance

Soybean (*Glycine max* (L.) Merr*.*) is the most important legume in the world. However, the rapid expansion of its cultivated areas has created new ecological niches for many pathogens. Among them, *Phytophthora sojae* (Kaufmann and Gerdemann) ranks as one of the most damaging soybean pests in the world. The most common method to control it is the introgression of resistance genes termed *Rps* (Resistance to *P. sojae*) into elite cultivars. This imposes a high selection pressure on *P. sojae* leading to the development of new virulent pathotypes. Consequently, more durable sources of resistance are needed to manage *P. sojae*. A complementary approach resides in the exploitation of quantitative trait loci (QTL) associated with partial resistance (PR) which has been found to be more durable and effective against a broad spectrum of pathotypes (Karhoff et al., 2019). Several QTLs for PR of soybean against *P. sojae* have already been reported and are listed on SoyBase (Grant *et al.*, 2010). However, limited information is available about the precise nature and role of genes within those QTLs.

To exploit PR efficiently, an in‐depth characterization of the genetic regions involved is essential. Recent advances in high throughput genotyping by sequencing (GBS) techniques exploiting next generation sequencing (NGS) technologies provide abundant genome‐wide SNPs at low cost allowing precise mapping. These NGS advancements have also revolutionized transcriptome profiling (RNA‐seq), making possible the analysis of differentially expressed genes located within QTLs, an approach that has been proposed as a critical validation tool for candidate genes. Moreover, RNA‐seq, as a genotyping tool, represents an alternative reduced‐representation approach focusing on protein‐coding regions (Scheben *et al.*, 2017).

One of the main challenges in the study of PR against *P. sojae* has been the lack of reliable methods to precisely characterize the phenotypes. Indeed, the most common assays, the layer test and/or the tray test, provide mainly qualitative estimates of PR, which can be biased in the presence of *Rps* genes (Karhoff et al*.*, 2019). To overcome these constraints, a hydroponic assay, developed by Lebreton *et al. *(2018), reproduces the key steps of the soybean–*P. sojae* interaction and allows the simultaneous inoculation of isolates covering all pathotypes thus eliminating the possible effect of *Rps* genes.

Sources of horizontal resistance against *P. sojae* are limited, and the problem is accentuated in Canada, where early maturity soybean varieties are required because of the short growing season. For this reason, the early maturity line PI 449459 reported to exhibit a high level of PR represented a rare opportunity. By using optimized GBS and the new phenotyping approach, the present study aimed to identify QTLs conferring PR using a recombinant inbred line (RIL) population derived from early maturity parents differing for PR. In order to define with greater resolution the putative genes involved in PR within QTLs, an RNA‐seq approach (BioProject ID: PRJNA574764) coupled with bioinformatic prediction tools were exploited to detect variation in gene expression and/or sequences to identify the most relevant candidate genes linked to PR.

Evaluation of the F_5:6_ RILs using the hydroponic assay coupled with a mixed inoculum carrying pathotypes to all common *Rps* genes provided a wide spectrum of responses going from plant death to severe to low root rot and to near absence of symptoms (Figure 1a). This response could be quantified using a single variable, the corrected dry weight (CDW; Stewart and Robertson, 2012), amenable to QTL analysis. When CDW (dry weight of inoculated vs control) of the RILs was measured at 21 dpi, a wide variation of phenotypes was obtained (Figure 1b).

**Figure 1:**
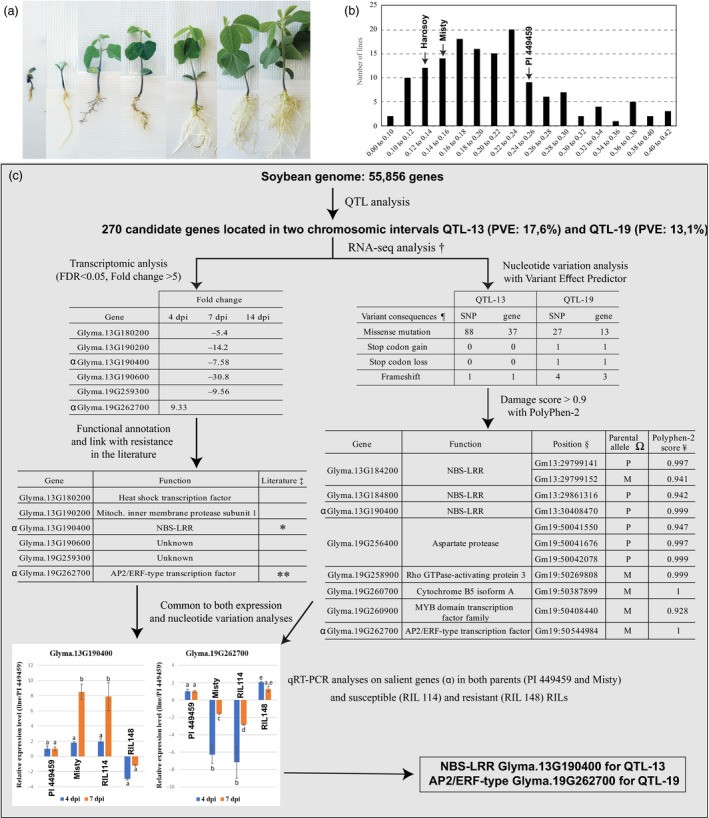
Quantitative trait loci, transcriptome and nucleotide variation analyses of partial resistance to *Phytophthora sojae* in a soybean population of 147 F_5:6_ recombinant inbred lines derived from the cross PI 449459 x Misty. (a) Range of PR phenotypes in soybean plants at 21 dpi following infection with a mixed inoculum of *P. sojae* (pathotypes 1a, 1b, 1c, 1d, 1k, 3a, 6 and 7) in hydroponics. (b) Frequency distribution of corrected total dry weight (CDW) values of soybean plants at 21 dpi from the RIL population. Harosoy was used as susceptible control. c) Pipeline used for the prioritization of candidate genes. †Summary of analyses of expression and nucleotide variation using RNA‐seq data for PI 449459 and Misty under infection with *P. sojae*. α qRT‐PCR analyses confirmed differential expression of *Glyma.13G190400* and *Glyma.19G262700* in both parental lines and RILs presenting susceptible (RIL114) and resistant (RIL148) phenotypes. ‡*McHale et al (2006) and Rasoolizadeh et al (2018), **Jisha et al (2015) and Phukan et al (2017). ¶Variant consequences were identified with Variant Effect Predictor (https://plants.ensembl.org/Glycine_max/Tools/VEP). § SNP positions were identified using the Fast‐WGS bioinformatic pipeline with RNA‐seq data of PI 449459 and Misty under infection with *P*. *sojae*. Ω P and M correspond to PI 449459 and Misty, respectively. ¥ Damaging score of SNPs was predicted with PolyPhen‐2 (Polymorphism Phenotyping v2; http://genetics.bwh.harvard.edu/pph2/). The score of a variant was characterized as ‘possibly damaging’ (score> 0.5–0.9) or ‘probably damaging’ (score> 0.9) based on a scale of 0–1.

A GBS approach on the F5 progeny of the cross between PI 449459 and Misty yielded a total of 1078 non‐redundant SNPs. The set of SNPs was subsequently used to construct a linkage map covering 2300 cM (over 93 % of the reference map) across 24 linkage groups representing 20 chromosomes with a marker density of one marker every 2.1 cM. Inclusive composite interval mapping using QTL IciMapping V 4.1 identified two QTLs inherited from PI 449459 and associated with PR to *P. sojae* in our RIL population. They were located on chromosomes 13 and 19 (designated QTL‐13 and QTL‐19) explaining 17.6% and 13.1% of the phenotypic variance, respectively (Figure 1c). The confidence intervals (defined using a one‐unit decrease of the peak LOD score) were mapped between 96.5 and 100.5 cM and were flanked by markers Chr13:28842184 and Chr13:30776191 (the nomenclature of markers is chromosome: physical position (bp)) for QTL‐13, and were mapped between 126.5 and 127 cM and were flanked by markers Chr19:50040258 and Chr19:50556102 for QTL‐19. The QTL‐13 and QTL‐19 intervals contained a total of 204 and 66 candidate genes, respectively. In order to reduce the number of candidate genes, the expression of these genes in response to infection and the predicted functional impact of nucleotide variants located within their coding regions were both investigated.

An RNA‐seq strategy to compare the different expression patterns of genes underlying the QTLs was performed by sequencing the transcriptomes of the resistant and susceptible parents, under both infected and control conditions. We focused on genes showing a > 5‐fold change in expression specific to *P. sojae* infection between the resistant and susceptible parents, and this analysis yielded four and two differentially expressed genes (DEGs) after infection for QTL‐13 and QTL‐19, respectively (Figure 1c).

In parallel, the sequence data from the RNA‐seq libraries were used to identify mutations inducing a modification in peptide sequence resulting in altered protein function. The effect of the SNPs was determined with the Variant Effect Predictor bioinformatic tool (Ensembl.org). One hundred and fifteen mis‐sense mutations, including two that induced a gain/loss of a stop codon and five inducing a frameshift, were found in 52 genes (Figure 1c). The analysis was further refined by examining SNPs predicted to modify the folding of proteins (score > 0.9) using PolyPhen‐2 (Kono *et al.*, 2018). This analysis uncovered 11 SNPs located in three and five candidate genes for QTL‐13 and QTL‐19, respectively.

Interestingly, NBS‐LRR *Glyma.13G190400* and AP2/ERF‐type transcription factor *Glyma.19G262700* were the only ones identified by both expression and nucleotide variation analyses making them extremely promising candidate genes for further functional characterization studies of PR to *P. sojae*. The RNA‐seq results for these genes were confirmed by qRT‐PCR with parental lines and RILs presenting susceptible and resistant phenotypes. AP2/ERF‐type transcription factors have been reported as playing a critical role in tolerance to biotic stress in several economically important crops such as rice, wheat, barley and soybean, leading Jisha *et al. *(2015) and Phukan *et al. *(2017) to recommend their use in breeding programmes. In our study, the AP2/ERF‐type transcription factor *Glyma.19G262700* was up‐regulated 9‐fold in the resistant genotype during the early stage of the infection process (4 dpi) which is consistent with a role in resistance as reported in the studies mentioned above. Also, of interest, *Glyma.13G190400* was expressed 7‐fold more in the susceptible genotype, which may favour a positive outcome for the pathogen (McHale *et al.*, 2006). Consistent with our results, Rasoolizadeh *et al. *(2018) observed a higher expression of NBS‐LRRs in a compatible interaction soybean–*P. sojae*.

In conclusion, this study proposes an integrated approach, exploiting a new phenotyping procedure, RNA‐seq analyses and SNP variants of predicted functional impact, to discriminate and prioritize high‐value candidate genes modulating complex quantitative traits such as those defining PR during plant–pathogen interactions.

## Conflict of interests

All authors declare no conflict of interest.
